# Estimating the Prevalence of Sexual Function Problems: The Impact of Morbidity Criteria

**DOI:** 10.1080/00224499.2015.1089214

**Published:** 2015-11-25

**Authors:** Kirstin R. Mitchell, Kyle G. Jones, Kaye Wellings, Anne M. Johnson, Cynthia A. Graham, Jessica Datta, Andrew J. Copas, John Bancroft, Pam Sonnenberg, Wendy Macdowall, Nigel Field, Catherine H. Mercer

**Affiliations:** ^a^ Centre for Reproductive and Sexual Health, London School of Hygiene and Tropical Medicine; ^b^ Dept of Infection and Population Health, University College London; ^c^ Dept of Psychology, University of Southampton; ^d^ Dept of Psychiatry, University of Oxford

**Keywords:** Prevalence, DSM-5, sexual function problems, morbidity criteria, classification

## Abstract

Establishing the clinical significance of symptoms of sexual dysfunction is challenging. To address this, the fifth edition of the Diagnostic and Statistical Manual of Mental Disorders (DSM-5) introduced two new morbidity criteria (duration and symptom severity) to the existing criteria of distress. This study sought to establish the impact of these three criteria on the population prevalence of sexual function problems. The data come from a national probability survey (Natsal-3) and are based on 11,509 male and female participants aged 16–74, reporting at least one sexual partner in the past year. The key outcomes were: proportion of individuals reporting proxy measures of DSM-5 problems, and the proportion of those meeting morbidity criteria. We found that among sexually active men, the prevalence of reporting one or more of four specific sexual problems was 38.2%, but 4.2% after applying the three morbidity criteria; corresponding figures for women reporting one or more of three specific sexual problems, were 22.8% and 3.6%. Just over a third of men and women reporting a problem meeting all three morbidity criteria had sought help in the last year. We conclude that the DSM-5 morbidity criteria impose a focus on clinically significant symptoms.

## Introduction

The challenge of distinguishing mild difficulties from clinical dysfunction has troubled psychiatry for many years (Mitchell & Graham, 2008; Wakefield, 1992). In the field of sexual dysfunction, this issue has become critical (Balon, Segraves, & Clayton, 2007), with the development of pharmacological treatments prompting concerns about the medicalization of sexual problems (Moynihan, 2010; Tiefer, 2012). Mild and transient sexual function problems are sufficiently common (Laumann, Paik, & Rosen, 1999; Mitchell et al., 2013) to be considered normal. The use of morbidity criteria—such as personal distress—to differentiate transient problems from dysfunction has generated significant debate (Hendrickx, Gijs, & Enzlin, 2013; Mitchell & Graham, 2008). The application of morbidity criteria in epidemiological research has been inconsistent, detracting from efforts to accurately assess prevalence, and leading to calls for more systematic measurement of severity (Derogatis & Burnett, 2008).

The Diagnostic and Statistical Manual of Mental Disorders (DSM American Psychiatric Association [APA], 2013) provides a standard and internationally recognized system for classifying sexual dysfunction. In the recently published fifth edition (DSM-5), two new conditions for morbidity were added to the existing distress criterion stipulated in DSM-IV-TR. There is now a requirement, across all diagnoses, that symptoms have persisted for a minimum duration of approximately six months; have been experienced in almost all or all (approximately 75%—100%) sexual encounters or have been persistent/recurrent; and have caused the individual clinically significant distress. The changes were specifically designed to improve precision, “reduce likelihood of overdiagnosis” and “distinguish transient sexual difficulties from more persistent sexual dysfunction” (APA, 2013, p. 809–816). The diagnostic categories were also extensively revised (see Box 1), reflecting a move away from the conceptualization of sexual response as a linear progression, essentially similar for women and men, towards recognition of substantial inter-personal variation without a single underlying model (Carvalheira, Brotto, & Leal, 2010; Graham, 2015; Sand & Fisher, 2007). Where the previous version (DSM-IV-TR) referred to “psychophysiological changes” and the “sexual response cycle” (APA, 2000; p. 261), in DSM-5 sexual dysfunction is defined as “a clinically significant disturbance in a person’s ability to respond sexually or to experience sexual pleasure” (American Psychiatric Association, 2013, p. 423). Furthermore, DSM-IV TR included subtypes “due to psychological factors” and “due to combined factors” (both psychological factors and general medical condition are etiological factors), but DSM-5 now stipulates a wider range of “associated features” to be considered during assessment and treatment. These are: “1) partner factors; 2) relationship factors; 3) individual vulnerability factors, psychiatric comorbidity or stressors; 4) cultural or religious factors; and 5) medical factors relevant to prognosis, course, or treatment” (APA, 2013, p. 423).

The changes to morbidity criteria have met with considerable opposition (Balon & Clayton, 2014; Clayton, DeRogatis, Rosen, & Pyke, 2012; Sungur & Gündüz, 2014). The chief criticism has been that “raising the bar” (Clayton et al., 2012, p. 2040) for a diagnosis will exclude individuals with dysfunction from treatment. The DSM-5 Sexual Dysfunctions subworkgroup has countered this criticism (Binik, Brotto, Graham, & Segraves, 2010; Graham, Brotto, & Zucker, 2014), citing the empirical basis for the DSM-5 criteria and pointing to the fact that previous versions of the DSM were criticized for poorly operationalized criteria (Binik et al., 2010). An explicit purpose of introducing morbidity criteria was to reduce the number of false positives (Sungur & Gündüz, 2014) and to provide more clinically useful thresholds (Binik et al., 2010). Some critics, however, have argued that the changes will “create havoc in the entire area of sexual dysfunction” (Balon & Clayton, 2014, p. 1227). On the other hand, narrower evidence-based criteria may actually protect patients by preventing healthy individuals with transient problems from being labeled as sick (Moynihan, 2010). However, potential loss of sensitivity with use of these more stringent criteria has not yet been investigated.

Given this debate, it is important to understand the impact of these morbidity criteria on the population prevalence of sexual dysfunction. This is not yet known because, to date, no study has employed all three morbidity criteria. In Britain’s third National Survey of Sexual Attitudes and Lifestyles (Natsal-3) we used different symptom measures but similar morbidity criteria to the DSM-5, enabling us to show the effect on prevalence estimates of sexual function problems and extent of overlap between problems when all three morbidity criteria are applied. We also used our survey data to investigate the empirical evidence for another key change in DSM-5: the introduction of the broader range of associated features relevant to assessment/treatment. Finally, we investigated the proportion of those reporting morbid difficulties who had sought professional help.

## Method

### Participants and Procedure

Natsal-3 is a stratified probability sample survey of 15,162 men and women aged 16–74 years in Britain, interviewed between September 2010 and August 2012. We used a multi-stage, clustered, and stratified probability sample design and participants were interviewed using a combination of computer-assisted face-to-face and self-interview (CASI) for the more sensitive questions. The survey instrument underwent thorough cognitive testing and piloting (Gray & Nicholson, 2009). After weighting to adjust for unequal probabilities of selection, the Natsal-3 sample was broadly representative of the British population as described by 2011 Census figures (Erens et al., 2013).

The estimated response rate was 57.7%, while the co-operation rate was estimated at 65.8% (of all eligible addresses contacted). Details of the survey methodology are published elsewhere (Erens et al., 2013; Mercer et al., 2013). Natsal-3 was approved by the NRES Committee South Central—Oxford A (Ref: 10/H0604/27). Participants provided oral informed consent for interviews.

### Outcome Measures

Participants reporting at least one sexual partner^1^ ^1^Defined as one person with whom they had had sex on at least one occasion, where sex is defined as vaginal intercourse, oral sex or anal sex. in the past year were classified as sexually active and asked whether they had experienced any of a list of eight difficulties with their sex life lasting 3 months or longer in the past year. If they reported a problem they were then directed to three further items asking how long they had experienced the problem (options: at least 3 months but less than 6 months, at least 6 months but less than a year, at least a year but less than 5 years, 5 years or longer); how often the symptoms occurred (options: always, very often, sometimes, not very often); and how they felt about the problem (options: not at all distressed, a little distressed, fairly distressed, very distressed). Several of the problems we assessed, including painful sex in men, and vaginal dryness in women, do not have DSM-5 diagnoses and so were excluded from our analyses. **Box 1** summarises the DSM-5 diagnostic criteria alongside the Natsal-3 survey items and morbidity criteria. We use the term “morbid sexual function problem” to refer to a problem meeting all three DSM-5 morbidity criteria. We assessed the proportion of individuals with one or more “morbid” problem who had sought professional help. This included family doctor, sexual health/genito-urinary medicine/STI clinic, psychiatrist or psychologist, relationship counsellor or other type of clinic or doctor.

We explored associations between reporting one or more problem(s) meeting all three morbidity criteria and a range of factors usually considered during a clinical assessment to support diagnosis and inform treatment. Items from the survey were selected to be consistent with five groups of associated features described in DSM-5 as being potentially relevant to etiology and/or treatment. These were: partner factors; relationship factors; individual vulnerability factors, including psychiatric co-morbidity, history of abuse, and unemployment; cultural or religious factors; and medical factors relevant to etiology and treatment (APA, 2013).

### Statistical Analysis

All analyses were done using the complex survey functions of STATA (version 12; StataCorp LP, College Station, Texas) to account for the weighting, clustering, and stratification of the data. Analysis was restricted to men and women reporting at least one sexual partner (of either gender) in the past year. We present descriptive statistics for reporting of sexual function problems meeting DSM-5 morbidity criteria and tested for associations with age and gender differences using the Chi square statistic. We used age-adjusted logistic regressions to examine the associations between reporting one or more morbid sexual function problem and the five groups of factors. We also examined associations with each morbid sexual function problem separately to then assess whether associations were broadly consistent across problems or not.

## Results

The demographic characteristics of the total Natsal-3 sample (n = 15,162) are described elsewhere (Mercer et al., 2013). For this study, we used data from sexually-active participants (n = 4,840 men and n = 6,669 women) defined as those who reported one or more sexual partner in the past year (Table 1).

**Table 1.** Sexually active population demographics

	Men		Women	
*Unweighted, weighted denominators*	*4840, 5975*		*6669, 5755*	
	Percent	95% C.I.	Percent	95% C.I.
Age group				
16–24	15.7%	(14.7–16.7)	16.0%	(15.2–16.9)
25–34	20.7%	(19.5–21.9)	21.6%	(20.7–22.6)
35–44	21.7%	(20.2–23.3)	22.4%	(21.2–23.7)
45–54	19.9%	(18.4–21.3)	20.6%	(19.3–22.0)
55–64	14.2%	(13.0–15.5)	13.1%	(12.1–14.2)
65–74	7.8%	(7.0–8.7)	6.2%	(5.5–6.9)
Marital status				
Married or civil partnership	54.7%	(53.1–56.3)	55.2%	(53.7–56.6)
Cohabitation	14.7%	(13.6–15.8)	14.2%	(13.2–15.2)
Previously married	6.0%	(5.4–6.6)	8.3%	(7.6–9.1)
Single and never married	24.6%	(23.3–25.9)	22.3%	(21.3–23.4)
Ethnic origin				
White	88.6%	(87.4–89.7)	89.5%	(88.5–90.3)
Mixed	1.6%	(1.2–2.0)	2.0%	(1.7–2.4)
Asian or Asian British	5.6%	(4.8–6.5)	4.4%	(3.8–5.0)
Black or Black British	3.2%	(2.6–4.0)	3.1%	(2.6–3.7)
Other	1.0%	(0.7–1.5)	1.1%	(0.8–1.4)
Self-defined sexual identity				
Heterosexual/straight	97.3%	(96.7–97.8)	97.1%	(96.6–97.5)
Gay/lesbian	1.5%	(1.2–2.0)	1.1%	(0.9–1.4)
Bisexual	1.1%	(0.8–1.5)	1.6%	(1.3–1.9)
Other	0.1%	(0.0–0.2)	0.2%	(0.1–0.4)
National Statistics Socio-Economic Classification				
Managerial and professional occupations	38.8%	(37.1–40.5)	33.7%	(32.4–35.1)
Intermediate occupations	17.3%	(16.0–18.7)	21.1%	(19.9–22.2)
Semiroutine and routine occupations	32.3%	(30.7–33.9)	26.9%	(25.7–28.1)
Never worked and long-term unemployed	4.4%	(3.8–5.1)	10.9%	(10.1–11.9)
Full-time students	7.2%	(6.5–8.1)	7.4%	(6.7–8.1)
Quintile of Index of Multiple Deprivation^a^				
1 [least deprived]	21.4%	(19.9–23.0)	21.0%	(19.6–22.5)
2	21.2%	(19.6–22.8)	21.0%	(19.5–22.5)
3	19.6%	(18.1–21.1)	19.4%	(18.0–20.8)
4	19.8%	(18.3–21.4)	19.7%	(18.4–21.2)
5 [most deprived]	18.0%	(16.6–19.5)	18.9%	(17.6–20.2)

^a^ A multidimensional measure of area (neighbourhood)-level deprivation based on the participant’s postcode; Index of Multiple Deprivation scores for England, Scotland, and Wales were adjusted before assignment to quintiles by use of a method by Payne and Abel (2012).

**Figure 1.**
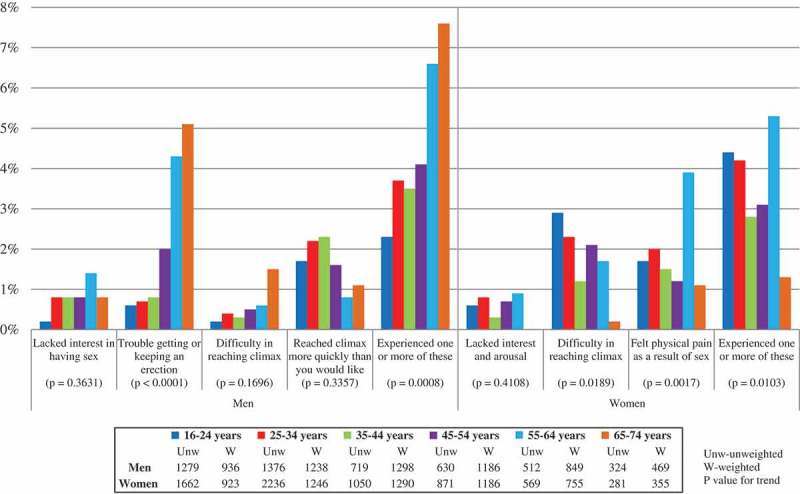
Prevalence of sexual function problems meeting DSM-5 morbidity criteria in the last year by gender and age group, among the sexually active participants.

Table 2 shows the proportion of sexually active men and women reporting sexual function problems lasting three months or more in the last year, as well as the proportion reporting problems meeting DSM-5 morbidity criteria. Among sexually active men, the one-year population prevalence estimates of individual sexual function problems lasting three months or more ranged from 9.2% (difficulty reaching climax), through 12.9% (getting and keeping an erection) to 15% (lacking interest in sex and reached climax more quickly than you would like). For all four problems the proportions fulfilling all three morbidity criteria were much lower, with difficulty reaching climax and lacking interest in sex showing sharper declines than the other problems, to 0.5% and 0.8% respectively. For example, a third of men reporting lack of interest had frequent symptoms, but less than 15% reported being distressed about it; and over 40% of men who had difficulty reaching climax had experienced the problem for six months or more, but only 16.3% experienced frequent symptoms. Distress was approximately twice as common in men reporting trouble getting or keeping an erection than in the other problems. In all, 11.1% of men reporting one or more problem met all three morbidity criteria: fewer than 6% of men reporting lack of interest in sex and fewer than 6% of men reporting difficulty reaching climax; 11.6% of men reporting reaching climax too quickly; and 14.1% of men reporting erectile difficulties. Thus, after applying morbidity criteria, erectile difficulties and reaching climax too quickly became the most commonly reported problems (1.8% and 1.7% of all sexually active men, respectively). In total, 4.2% of sexually active men reported one or more sexual function problem meeting DSM-5 morbidity criteria in the past year. Considering morbid sexual function problems, only difficulty getting/keeping an erection was strongly associated with older age (Figure 1), increasing from 0.6% of men aged 16–24 to 5.1% of men aged 65–74 (p < 0.0001).

**Table 2.** Percentage of sexually active men and women reporting sexual function problems for at least three months in the last year and proportion reporting problems meeting DSM-5 morbidity criteria

	Of those reporting the problem					
	Population % reporting problem	Lasting 6 months or more	Always/very often symptomatic	Fairly/very distressing	Meets all 3 DSM-5 criteria	Population % meeting DSM-5 morbidity criteria
**Men (Unw 4840; W 5975)**						
Lacked interest in having sex	15.0%	35.1%	32.2%	14.4%	5.2%	0.8%
95% C.I.	(13.9–16.2)	(31.0–39.4)	(28.3–36.3)	(11.8–17.5)	(3.6–7.5)	(0.5–1.1)
Trouble getting or keeping an erection	12.9%	64.2%	26.7%	40.9%	14.1%	1.8%
95% C.I.	(11.8–14.0)	(59.6–68.4)	(22.8–30.8)	(36.5–45.5)	(11.3–17.5)	(1.4–2.3)
Difficulty in reaching climax	9.2%	43.7%	16.3%	17.3%	5.5%	0.5%
95% C.I.	(8.3–10.1)	(38.6–49.0)	(12.9–20.4)	(13.6–21.7)	(3.5–8.5)	(0.3–0.8)
Reached climax more quickly than you would like	14.9%	53.3%	29.9%	22.2%	11.6%	1.7%
95% C.I.	(13.7–16.2)	(48.9–57.7)	(26.2–33.9)	(18.8–25.9)	(9.0–14.7)	(1.3–2.2)
Experienced one or more of these problems	38.2%	53.0%	32.8%	24.9%	11.1%	4.2%
95% C.I.	(36.6–39.8)	(50.3–55.7)	(30.4–35.3)	(22.7–27.2)	(9.5–12.9)	(3.6–5.0)
**Women (Unw 6669; W 5755)**						
Lacked interest and arousal	6.5%	55.8%	33.1%	23.8%	9.1%	0.6%
95% C.I.	(5.9–7.2)	(50.3–61.1)	(27.8–38.8)	(19.6–28.6)	(6.4–12.8)	(0.4–0.9)
Difficulty in reaching climax	16.3%	52.9%	39.0%	22.0%	11.6%	1.9%
95% C.I.	(15.3–17.3)	(49.5–56.3)	(35.7–42.5)	(19.4–24.8)	(9.7–13.8)	(1.6–2.3)
Felt physical pain as a result of sex	7.4%	62.1%	50.8%	45.5%	25.0%	1.9%
95% C.I.	(6.7–8.3)	(57.1–66.8)	(45.6–56.1)	(40.5–50.5)	(20.7–29.8)	(1.5–2.3)
Experienced one or more of these problems	22.8%	55.5%	43.6%	28.8%	16.0%	3.6%
95% C.I.	(21.7–24.0)	(52.5–58.4)	(40.6–46.6)	(26.3–31.4)	(14.0–18.2)	(3.2–4.2)

Unw = unweighted denominator; W = Weighted denominator

Among sexually active women, the one-year population prevalence estimates of sexual function problems lasting three months or more ranged from 6.5% (lack of interest and arousal) to 16.3% (difficulty reaching climax). As with men, the proportion of women fulfilling all three DSM-5 morbidity criteria was much lower for the three problems included in the analysis. The decline was less among women reporting pain, of whom 25% met all three morbidity criteria, compared with 9.1% of women reporting lack of interest and arousal and 11.6% of women reporting difficulty reaching climax. In particular, the proportion reporting distress was twice as high among women reporting pain compared with the other two problems. In all, 16.0% of women reporting one or more problem met all three morbidity criteria. After applying morbidity criteria, difficulty reaching climax and pain were the most commonly reported sexual problems, each reported by 1.9% of women, followed by lack of interest and arousal, reported by 0.6% of women. In all, 3.6% of sexually active women reported one or more problems meeting DSM-5 morbidity criteria in the past year. We found significant variation by age for difficulty reaching climax (p = 0.02) and pain (p = 0.002, but not for lack of interest and arousal (Figure 1). There was no linear increase in age for reporting one or more morbid sexual function problem; rather, problems were most common in younger age groups (16–34) and in later mid-life (55–64), declining significantly after age 64.

## Overlap Between Severe Sexual Function Problems

Table 3 (a and b) shows the overlap in reported morbid sexual function problems among sexually active men and women. Among men who met criteria for morbid lack of interest in sex, approximately a third (35.9%) also reported another of the morbid sexual function problems. Although numbers are small, fewer men reporting erectile difficulties (16.5%) and reaching climax more quickly than they would like (11.0%) also reported another morbid problem. Among women reporting morbid lack of interest and arousal, most (71.6%) also reported another morbid problem; specifically, 57.8% also reported morbid orgasmic difficulty and 33.4% also experienced morbid pain. By contrast, only a minority of women reporting difficulty reaching climax (26.1%) and pain (18.8%) also reported another morbid problem.

**Table 3a:** Overlap between reported sexual function problems meeting DSM-5 morbidity criteria: Sexually active men

	Percentage also experiencing:										
Men	Lacked interest in having sex		Trouble getting or keeping an erection		Difficulty in reaching climax		Reached climax more quickly than you would like		Any of the other problems in table		Denominators
	%	95% CI	%	95% CI	%	95% CI	%	95% CI	%	95% CI	Unw W
Lacked interest in having sex	-	-	12.4%	(5.2–26.8)	15.5%	(6.3–33.3)	17.4%	(7.4–35.7)	35.9%	(20.6–54.7)	37, 46
Trouble getting or keeping an erection	5.4%	(2.3–11.9)	-	-	10.8%	(5.4–20.3)	3.0%	(1.0–8.5)	16.5%	(9.9–26.2)	82, 108
Difficulty in reaching climax*	NA	-	NA	-	-	-	NA	-	NA	-	22, 30
Reached climax more quickly than you would like	7.9%	(3.3–17.4)	3.2%	(1.1–8.9)	1.5%	(0.2–10.1)	-	-	11.0%	(5.6–20.6)	82, 103

* Numbers too small to permit analysis

**Table 3b.** Overlap between reported sexual function problems meeting DSM-5 morbidity criteria: Sexually active women

	Percentage also experiencing:								
Women	Lacked interest and arousal in having sex		Difficulty in reaching climax		Felt physical pain as a result of sex		Any of the other problems in the table		Denominators
	%	95% CI	%	95% CI	%	95% CI	%	95% CI	Unw W
Lacked interest and arousal in having sex	-	-	57.8%	(40.1–73.6)	33.4%	(18.6–52.4)	71.6%	(54.4–84.2)	42, 34
Difficulty in reaching climax	18.1%	(11.5–27.5)	-	-	14.1%	(8.6–22.2)	26.1%	(18.2–35.9)	145, 109
Felt physical pain as a result of sex	10.7%	(5.8–18.7)	14.3%	(8.7–22.7)	-	-	18.8%	(12.2–27.8)	119, 107

### Factors Relevant to Clinical Assessment

Table 4 shows factors considered relevant to diagnosis and treatment of sexual disorders in DSM-5, and their association with reporting one or more morbid sexual function problem. Among women, there were significant associations with all factors except unemployment, religiosity and the attitude that ‘sex without love is OK’. The strongest associations were with feeling anxious during sex (OR 12.15; 95% C.I. 8.55–17.25), reporting vaginal dryness (7.19; 5.23–9.87), and reporting a health condition that affected sexual activity and enjoyment (7.44; 5.48–10.09). The sexual relationship was also important, in particular not sharing the same level of interest in sex (4.43; 3.07–6.39) and not sharing the same likes and dislikes (3.92; 2.54–6.03). When we analyzed associations with each individual problem (supplemental Table 1) we found they were broadly consistent across problems in the sense that there were no instances of strong associations in opposite directions. Qualitative assessment of odds ratios showed that lack of interest and arousal was more strongly associated than other individual problems with anxiety and with not sharing the same level of interest in sex as a partner; pain was more strongly associated with difficulty talking about sex; and difficulty reaching climax was more strongly associated with having a partner with sexual difficulties.

**Table 4.** Associations between sexual function problems meeting DSM-5 morbidity criteria and factors relevant to etiology and management

	Men						Women					
	%	95% C.I.	aAOR	95% C.I.	p-value	Denominators^a^	%	95% C.I.	aAOR	95% C.I.	p-value	Denominators^a^
Overall	4.2%	(3.6–5.0)	-	-	-	4840, 5975	3.6%	(3.2–4.2)	-	-	-	6669, 5755
Age group					0.0002						0.0171	
16–24	2.3%	(1.6–3.4)	1.00			1279, 936	4.4%	(3.5–5.6)	1.00			1662, 923
25–34	3.7%	(2.6–5.1)	1.59	(0.95–2.64)		1376, 1238	4.2%	(3.3–5.3)	0.94	(0.67–1.33)		2236, 1246
35–44	3.5%	(2.5–5.1)	1.52	(0.91–2.55)		719, 1298	2.8%	(1.9–4.0)	0.61	(0.39–0.96)		1050, 1290
45–54	4.1%	(2.6–6.2)	1.77	(1.00–3.13)		630, 1186	3.1%	(2.1–4.6)	0.68	(0.42–1.10)		871, 1186
55–64	6.6%	(4.7–9.2)	2.93	(1.74–4.94)		512, 849	5.3%	(3.6–7.7)	1.20	(0.74–1.93)		569, 755
65–74	7.6%	(5.0–11.6)	3.43	(1.89–6.24)		324, 469	1.3%	(0.5–3.3)	0.29	(0.11–0.75)		281, 355
PARTNER AND RELATIONSHIP FACTORS												
Partner had sexual difficulties past year					0.0676						0.0002	
No	3.8%	(3.0–4.7)	1.00			2431, 3454	3.2%	(2.6–3.9)	1.00			3726, 3498
Yes	6.1%	(4.1–8.9)	1.59	(0.97–2.61)		513, 763	6.4%	(4.6–8.8)	2.26	(1.47–3.48)		649, 719
Always easy to talk about sex with partner					0.0001						0.0057	
Yes	2.2%	(1.5–3.1)	1.00			1695, 1899	2.5%	(1.8–3.4)	1.00			1746, 1451
Else	5.2%	(4.4–6.2)	2.26	(1.50–3.42)		3123, 4050	4.0%	(3.5–4.7)	1.66	(1.16–2.38)		4907, 4289
Partner shares same interest level in sex					<0.0001						<0.0001	
Yes	3.1%	(2.4–4.0)	1.00			2270, 3233	2.0%	(1.5–2.6)	1.00			3211, 3064
No	7.7%	(5.7–10.3)	2.65	(1.75–4.02)		676, 988	8.3%	(6.7–10.3)	4.43	(3.07–6.39)		1166, 1155
Partner shares same sexual likes and dislikes					0.0001						<0.0001	
Yes	3.7%	(2.9–4.6)	1.00			2650, 3803	3.1%	(2.6–3.8)	1.00			4079, 3908
No	9.0%	(6.0–13.2)	2.65	(1.61–4.35)		296, 418	11.2%	(7.9–15.7)	3.92	(2.54–6.03)		297, 310
INDIVIDUAL VULNERABILITY												
Felt anxious during sex					<0.0001						<0.0001	
No	3.4%	(2.8–4.1)	1.00			4548, 5651	2.5%	(2.1–3.0)	1.00			6264, 5453
Yes	19.4%	(15.0–24.9)	7.46	(5.12–10.87)		292, 324	23.5%	(18.8–28.9)	12.15	(8.55–17.25)		405, 302
Experienced non-volitional sex, ever^b^					0.1269						<0.0001	
No	4.1%	(3.5–4.9)	1.00			4706, 5825	3.1%	(2.6–3.6)	1.00			5815, 5055
Yes	7.9%	(3.2–18.3)	2.09	(0.81–5.40)		71, 82	8.9%	(6.8–11.6)	3.10	(2.21–4.35)		684, 579
Unemployed last week					0.0198						0.8154	
No	3.6%	(2.9–4.4)	1.00			3277, 4307	3.7%	(3.1–4.4)	1.00			4001, 3597
Yes	6.0%	(4.6–7.7)	1.53	(1.07–2.19)		1560, 1666	3.6%	(2.8–4.4)	0.97	(0.73–1.29)		2662, 2152
Current depression (PHQ-2)^c^					<0.0001						<0.0001	
No	3.5%	(2.9–4.2)	1.00			4384, 5472	3.0%	(2.6–3.6)	1.00			5885, 5149
Yes	12.4%	(9.1–16.6)	4.17	(2.77–6.26)		449, 495	8.9%	(6.9–11.5)	3.12	(2.25–4.33)		780, 602
CULTURAL/RELIGIOUS FACTORS												
Religion important and practiced regularly					0.6125						0.1346	
No	4.2%	(3.5–4.9)	1.00			4481, 5453	3.8%	(3.3–4.4)	1.00			6047, 5100
Yes	5.2%	(3.1–8.6)	1.16	(0.65–2.07)		349, 506	2.4%	(1.3–4.3)	0.62	(0.33–1.16)		609, 644
Sex without love is OK					0.9122						0.1004	
No	4.5%	(3.4–5.8)	1.00			1586, 2097	3.2%	(2.5–4.0)	1.00			3291, 2968
Yes	4.2%	(3.4–5.1)	1.02	(0.72–1.45)		3233, 3852	4.1%	(3.4–5.0)	1.29	(0.95–1.76)		3347, 2761
People are under pressure to have sex					0.1817						0.0158	
No	3.7%	(2.8–4.9)	1.00			1737, 2176	2.6%	(1.9–3.6)	1.00			1761, 1485
Yes	4.6%	(3.8–5.6)	1.28	(0.89–1.85)		3039, 3708	4.1%	(3.5–4.8)	1.61	(1.09–2.37)		4817, 4185
MEDICAL FACTORS												
Number of self-reported chronic conditions^d^					<0.0001						<0.0001	
0	3.0%	(2.4–3.8)	1.00			3456, 3996	2.5%	(2.0–3.1)	1.00			4357, 3536
1	4.6%	(3.4–6.1)	1.39	(0.90–2.16)		920, 1302	4.5%	(3.5–5.8)	2.07	(1.47–2.92)		1544, 1405
2+	11.0%	(8.0–14.8)	3.35	(2.03–5.51)		464, 678	7.1%	(5.2–9.6)	3.90	(2.60–5.85)		767, 814
Health condition affecting sexual activity or enjoyment					<0.0001						<0.0001	
No	2.3%	(1.8–2.9)	1.00			4170, 5061	1.8%	(1.5–2.2)	1.00			5515, 4712
Yes	15.0%	(12.2–18.4)	6.89	(4.81–9.86)		656, 898	11.9%	(9.8–14.4)	7.44	(5.48–10.09)		1147, 1038
Medication that affected sexual activity last year					<0.0001						<0.0001	
No	3.3%	(2.8–4.1)	1.00			4492, 5513	2.9%	(2.5–3.5)	1.00			6170, 5318
Yes	15.2%	(11.4–19.9)	4.46	(2.96–6.72)		332, 444	12.4%	(9.4–16.2)	4.67	(3.27–6.69)		492, 431
Uncomfortably dry vagina					-						<0.0001	
No	-	-	-	-		-	2.3%	(1.9–2.7)	1.00			5920, 5010
Yes	-	-	-	-		-	13.0%	(10.4–16.1)	7.19	(5.23–9.87)		749, 746

aAOR = age-Adjusted Odds Ratio; PHQ-2 = Patient Health Questionnaire-2

^a^ Unweighted, weighted denominators

^b^ Defined as anyone having sex with you against your will after the age of 13 years

^c^ Two screening questions (scored 0–3 per question; defined here by a total score of 3 or more) assessed depressive symptoms (PHQ-2; Arroll, 2003; Arroll et al., 2010)

^d^ Includes arthritis, heart attack, coronary heart disease, angina, other forms of heart disease, hypertension, stroke, diabetes, broken hip or pelvis bone or hip replacement ever, backache lasting longer than 3 months, any other muscle or bone disease lasting longer than 3 months, depression, cancer, and any thyroid condition treated in the past year

Similar to women, there was a strong association among men between reporting one or more morbid sexual function problem and anxiety (OR 7.46; 95%CI 5.12–10.87) and with all three health factors (number of self-reported chronic conditions, health and medication affecting sexual activity and enjoyment in the past year), as well as with depression (4.17; 2.77–6.26) and three of the four ‘partner and sexual relationship’ variables. Unlike women, there was no association with agreeing that ‘people are under pressure to have sex’ (1.28; 0.89–1.85). The analysis of individual problems showed no instances of strong effects in opposite directions, again suggesting that associations are broadly consistent across problems (supplemental data Table 1). However, lacking interest in sex appeared more strongly associated than the other individual problems with partner and relationship factors; we also found that difficulty reaching climax was more closely associated than the other individual problems with attitudes to sex (disagreeing that “sex without love is ok” and that agreeing that “people are under pressure to have sex”) and that associations with health were less strong for reaching climax too quickly.

A second regression model to analyze associations with one or more sexual function problem, whether meeting morbidity criteria or not, showed similar results to Table 3 (data not shown).

### Seeking Professional Help

**Figure 2.**
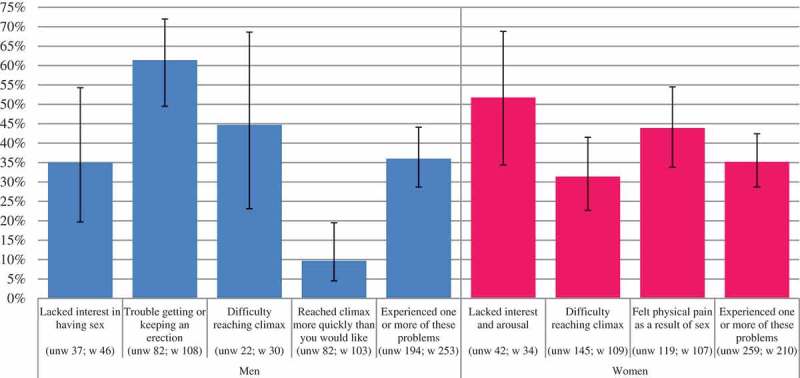
Proportion of men and women with sexual function problems meeting DSM-5 morbidity criteria who sought professional help in the last year.

Among men reporting one or more morbid sexual function problems (Figure 2), professional help was most commonly sought, by just over 60% of men, for trouble getting or keeping an erection. It was least commonly sought (under 10%), by men reporting difficulty with early climax. Across all morbid problems, just over a third of men had sought professional help about their sex life in the last year. Among women, help seeking in the last year was most common among those reporting a problem with interest and arousal (51.8% of women meeting all three morbidity criteria). Again, across all problems, just over a third of women had sought professional help in the past year.

## Discussion

DSM-5 represented a significant shift in the classification of sexual dysfunction, most notably in relation to the classification of female disorders, but also with regard to the level of morbidity required for diagnosis. Changes in DSM criteria clearly have a bearing on the measurement of disorders in epidemiological studies and these Natsal-3 data, to our knowledge, are the first to indicate the likely nature of these changes. In the sexually active British population, we found that among sexually active men, the prevalence of reporting one or more of four specific sexual problems was 38.2%, but 4.2% after applying the three DSM-5 morbidity criteria. Among sexually active women, the prevalence of reporting one or more of three specific sexual problems was 22.8%, but 3.6% after applying the three morbidity criteria. Of all men and women reporting one or more of the seven sexual function problems lasting three months or more in the last year, 11.1% of men and 16% of women reported at least one problem that met all three DSM-5 morbidity criteria.

The strength of our study is that it is based on a large sample with a wide age range and is representative of the British adult population (Erens et al., 2013; Mercer et al., 2013). We achieved a response rate in line with other major social surveys in Britain (Park, Clery, Curtice, Phillips, & Utting, 2012) and higher than many surveys of sexual dysfunction (Christensen et al., 2011; Laumann et al., 2006). A limitation is that we report the prevalence of sexual function problems approximating the DSM-5 criteria for morbidity, rather than the prevalence of clinical sexual disorders as classified by DSM-5. It is not feasible for cross-sectional surveys to provide sufficient clinical information for a definite diagnosis (Graham & Bancroft, 2005). For instance, the DSM-5 stipulates that if the sexual problem is attributable to a medical condition, then a diagnosis of sexual dysfunction is not given (American Psychiatric Association, 2013); it is not possible to ascertain such causality in a cross-sectional survey. Furthermore, there were notable differences in the definition of individual sexual problems in our study compared with the DSM-5. For instance, the new DSM-5 Female Sexual Interest//Arousal Disorder uses a polythetic approach, requiring three of six possible symptoms to be present. In Natsal-3 we approximated this classification by including women who reported both lack of interest and lack of excitement/arousal. The extent to which our combined category represents an adequate proxy for the new DSM-5 diagnostic criteria is unknown as there are not yet prevalence studies using the new polythetic approach; it is likely that including only women who report lack of interest AND lack of excitement/arousal gives rise to a lower prevalence estimate than requiring the presence of 3 out of 6 symptoms of FSIAD (either arousal or desire symptoms). Other Natsal-3 definitions were less strict than in DSM-5; for premature ejaculation we included all men who reported reaching a climax more quickly than they would like, whereas the DSM-5 diagnosis requires that men persistently experience ejaculation within 1 minute of vaginal penetration and before the individual wishes it. For these reasons we focus in this paper on the impact of DSM-5 morbidity criteria on prevalence of sexual function problems, rather than on the prevalence estimates themselves. Finally, the small number of participants meeting DSM-5 morbidity criteria limited our ability to explore associations with individual problems with sufficient accuracy.

As expected, our prevalence estimates using the DSM-5 morbidity criteria were lower than those of population studies adopting less stringent severity criteria (Christensen et al., 2011; Laumann, Glasser, Neves, & Moreira, 2009; Quinta Gomes & Nobre, 2014). We also found lower levels of overlap between different sexual problems than in previous studies (Quinta Gomes & Nobre, 2014; Fugl-Meyer & Fugl-Meyer, 2002). Overlap between diagnostic categories has previously been a source of criticism of the DSM classification because it leads to increased complexity and reduced clinical utility (First, 2005). It is difficult to ascertain whether overlap is an artifact of the classification system or true co-morbidity (Maj, 2005), but in terms of clinical utility, reduced overlap appears welcome.

An admirable feature of the DSM-5 classification of sexual dysfunction is the recognition of biological, psychological, and social factors (termed “associated features” in DSM-5) in understanding etiology and informing treatment decisions. Of the factors tested, our data provided strong support for their inclusion in DSM-5 since, with few exceptions, they were significantly associated with reporting morbid sexual function problems. Moreover, since the associations held for reports of all problems, regardless of morbidity, these factors appear to be relevant, whether or not problems meet morbidity criteria. Our findings support previous research demonstrating a strong contributing role of both depression and anxiety (McCabe et al., 2010), and for aspects of the sexual relationship such as compatibility (Witting et al., 2008). We also identified some interesting gender differences: unemployment was associated with male but not female dysfunction, and experience of non-volitional sex was more strongly associated among women (although the lack of an association in men may be in part be due to small numbers). We found no association with religiosity but this is possibly because the complexity of the construct and diversity of influence across individuals makes it difficult to capture in a brief survey item.

### Implications for Research and Practice

If we are to address the inconsistent measurement of severity in epidemiological studies, then adoption of standardized criteria, such as those in DSM-5, seems an appropriate way forward. Studies adopting these criteria should expect that of those reporting a sexual function problem, around 1 in 10 men and 1 in 6 women would meet all three morbidity criteria. Although logically, the morbidity criteria exclude those reporting milder symptoms and include those reporting more severe symptoms, a clinical study of specificity/sensitivity is required to judge the extent to which the criteria result in false negatives and false positives.

Previous surveys have emphasized the pervasiveness of sexual dysfunction, for example, the oft-cited, though highly criticized, estimates of 43% for women and 31% for men (Laumann et al., 1999). The close involvement of the pharmaceutical industry in the measurement and classification of sexual dysfunction has attracted significant criticism (Marshall, 2009; Moynihan, 2010; Tiefer, 2006). The industry has been accused of ‘disease mongering,’ including tactics such as encouraging mild symptoms to be viewed as severe and using prevalence estimates to suggest large numbers of people affected, with the purpose of creating demand for pharmacological intervention (Moynihan, Heath, & Henry, 2002; Payer, 1992; Tiefer, 2006). Prevalence studies have also been conducted against a background of increased labeling and medicalization of behavior in general, and concomitant rise in medication to treat behavioural disorders (Spence, 2012). Applying more stringent morbidity criteria certainly gives rise to lower estimates and the considerable size of the reduction suggests that previous prevalence figures may have over-estimated the scale of the problem. However, our data still suggest significant numbers of affected individuals: 8.9 million adults in the US and 1.8 million adults in the UK.^2^ ^2^Estimate based on 2014 census estimates for adults aged 16-74. Data for UK from Office for National Statistics (2015), and for US from US Census Bureau, Population Division (2015). These estimates are conservative since they are based on the sexually active population and omit those who may not be having sex because of sexual problems. In the same study (Mitchell et al., 2013) we found that among those who were ever sexually experienced (n = 1,034 men and n = 1,685 women), 21% of men and 17% of women reported avoiding sex because of a sexual difficulty, either their own or a partner’s. Of this group, it could be assumed that at least the same proportion as in this study (11% of men and 16% of women reporting any problem, among sexually active participants) met all three morbidity criteria for the problem they were avoiding but were not included in the overall prevalence estimates. This equates to 24 men and 46 women in our sample. Our data on help-seeking also suggest a high level of unmet need; almost two-thirds of men and women with morbid symptoms did not seek professional help for their sex life in the past year.

## Conclusion

The new DSM-5 morbidity criteria impose a focus on individuals who are experiencing persistent, frequent and distressing symptoms. Implementing these criteria in a population survey leads to much lower prevalence estimates and reduces overlap between problems. It is possible that prevalence estimates using insufficiently stringent morbidity criteria actually weaken arguments for resources by producing estimates that are not particularly credible (Balon, 2008) and by inducing inertia because the disease burden seems so large. The new DSM-5 morbidity criteria are welcome not only because they concentrate on those with clinically significant symptoms, but also because they suggest that it may be possible to address the disease burden caused by sexual problems, given sufficient commitment and resources.

**Box 1.** Criteria for sexual function problems: Comparison of Natsal-3 survey and DSM-5

DSM-5 diagnostic criteria: Verbatim extracts from DSM-5 (American Psychiatric Association, 2013, pp. 423‒450)	Natsal-3 survey item
**MORBIDITY CRITERIA**	
**Morbidity criteria for each difficulty** Symptoms experienced on almost all or all occasions (approx. 75–100%)* Symptoms must have been present for at least 6 months Symptoms cause clinically significant distress in the individual * Note that for Male Hypoactive Sexual Desire Disorder (HSDD) and Genito-Pelvic Pain/Penetration Disorder, symptoms should be ‘persistent or recurrent’ rather than occurring ‘always or almost always’	**Report if difficulty experienced for three months or more in last year. Items on morbidity asked for each difficulty endorsed** **Morbidity criteria for each difficulty**: *Symptoms occur ‘very often’ or ‘always’* *Experienced for at least 6 months* *Participant is ‘fairly distressed’ or ‘very distressed’ about the difficulty*
**FEMALE PROBLEMS**	
**Female Orgasmic Disorder**Either of the following symptoms: *Marked delay in, marked infrequency, or absence of orgasm*. *Markedly reduced intensity of orgasmic sensation*.	**Did not reach a climax (experience an orgasm) or took a long time to reach a climax despite feeling excited or aroused**
**Female Sexual Interest/Arousal disorder (FSIAD).** Diagnosis requires 3 or more of the following symptoms: *Absent/reduced interest in sexual activity*. *Absent/reduced sexual/erotic thoughts or fantasies*. *No/reduced initiation of sexual activity, and typically unreceptive to a partner’s attempts to initiate*. *Absent/reduced sexual excitement/pleasure during sexual activity on almost all or all (approximately 75%–100% of) sexual encounters (in identified situational contexts or, if generalized, in all contexts)*. *Absent/reduced sexual interest/arousal in response to any internal or external sexual/erotic cues (e.g., written, verbal, visual)*. *Absent/reduced genital or nongenital sensations during sexual activity on almost all or all (approximately 75%–100% of) sexual encounters (in identified situational contexts or, if generalized, in all contexts)*.	**Reported BOTH of these items**: **Lacked interest in having sex** **Felt no excitement or arousal during sex**
**Genito Pelvic Pain/Penetration disorder**.Persistent or recurrent difficulty with one or more of the following: *Having vaginal intercourse/penetration*. *Marked vulvovaginal or pelvic pain during vaginal intercourse or penetration attempts*. *Marked fear or anxiety either about vulvovaginal or pelvic pain in anticipation of, during, or as a result of vaginal penetration*. *Marked tensing or tightening of the pelvic floor muscles during attempted vaginal penetration*.	**Felt physical pain as a result of sex**
**MALE PROBLEMS**	
**Delayed ejaculation** Either of the following symptoms: *Marked delay in ejaculation* *Marked infrequency or absence of ejaculation*	**Did not reach a climax (experience an orgasm) or took a long time to reach a climax despite feeling excited or aroused**
**Erectile Disorder**: *At least one of the following*: *Marked difficulty in obtaining an erection during sexual activity*. *Marked difficulty in maintaining an erection until the completion of sexual activity*. *Marked decrease in erectile rigidity*.	**Had trouble getting or keeping an erection**
**Male Hyposexual Desire Disorder**: *Persistently or recurrently deficient (or absent) sexual/erotic thoughts or fantasies and desire for sexual activity*.	**Lacked interest in having sex**
**Premature (Early) Ejaculation**: *A persistent or recurrent pattern of ejaculation occurring during partnered sexual activity within approximately 1 minute following vaginal penetration and before the individual wishes it*.	**Reached climax (experienced an orgasm) more quickly than you would like**

## Supplementary Material

Supplemental Material
