# Supplementary material for: Estimating the Prevalence of Sexual Function Problems: The Impact of Morbidity Criteria
Source: J Sex Res. 2015 Nov 25;53(8):955–67. doi: 10.1080/00224499.2015.1089214 (PMC5044769; doi:10.1080/00224499.2015.1089214)
Supplement: Supplemental Material [file hjsr_a_1089214_sm9640.docx]

**Supplemental data for Online posting**

*Supplemental data table 1: Factors associated with reporting individual morbid sexual function problems among sexually active men and women aged 16 to 74*

|  | **Men** | | | | | | | | | | | | |
| --- | --- | --- | --- | --- | --- | --- | --- | --- | --- | --- | --- | --- | --- |
|  | **Lacked interest in having sex** | | | **Difficulty maintaining erection** | | | **Reached climax too slowly/not at all** | | | **Reached climax too quickly** | | |  |
|  | **aAOR** | **95% C.I.** | **p-value** | **aAOR** | **95% C.I.** | **p-value** | **aAOR** | **95% C.I.** | **p-value** | **aAOR** | **95% C.I.** | **p-value** | **Denominators** |
| **Age group** |  |  | 0.1654 |  |  | 0.0000 |  |  | 0.3787 |  |  | 0.3595 |  |
| 16-24 | 1.00 |  |  | 1.00 |  |  | 1.00 |  |  | 1.00 |  |  | 1279, 936 |
| 25-34 | 4.48 | (1.21-16.59) |  | 1.19 | (0.48-2.94) |  | 1.48 | (0.31-7.01) |  | 1.35 | (0.68-2.67) |  | 1376, 1238 |
| 35-44 | 4.49 | (1.15-17.57) |  | 1.40 | (0.52-3.79) |  | 1.43 | (0.26-7.77) |  | 1.37 | (0.69-2.69) |  | 719, 1298 |
| 45-54 | 4.21 | (0.82-21.57) |  | 3.41 | (1.39-8.36) |  | 2.23 | (0.47-10.55) |  | 0.94 | (0.39-2.23) |  | 630, 1186 |
| 55-64 | 7.64 | (1.83-31.96) |  | 7.59 | (3.32-17.38) |  | 2.53 | (0.47-13.76) |  | 0.45 | (0.15-1.29) |  | 512, 849 |
| 65-74 | 4.30 | (0.81-22.68) |  | 9.08 | (3.83-21.55) |  | 6.33 | (1.07-37.45) |  | 0.67 | (0.19-2.32) |  | 324, 469 |
| **PARTNER AND RELATIONSHIP FACTORS** |  |  |  |  |  |  |  |  |  |  |  |  |  |
| **Partner had sexual difficulties past year** |  |  | 0.2817 |  |  | 0.5084 |  |  | 0.8495 |  |  | 0.0566 |  |
| No | 1.00 |  |  | 1.00 |  |  | 1.00 |  |  | 1.00 |  |  | 2431, 3454 |
| Yes | 1.70 | (0.65-4.45) |  | 1.27 | (0.63-2.54) |  | 1.13 | (0.31-4.15) |  | 2.10 | (0.98-4.49) |  | 513, 763 |
| **Always easy to talk about sex with partner** |  |  | 0.0011 |  |  | 0.1758 |  |  | 0.1708 |  |  | 0.0029 |  |
| Yes | 1.00 |  |  | 1.00 |  |  | 1.00 |  |  | 1.00 |  |  | 1695, 1899 |
| Else | 4.10 | (1.76-9.55) |  | 1.50 | (0.83-2.69) |  | 2.26 | (0.70-7.26) |  | 2.76 | (1.42-5.39) |  | 3123, 4050 |
| **Partner shares same interest level in sex** |  |  | <0.0001 |  |  | 0.1049 |  |  | 0.6605 |  |  | 0.0006 |  |
| No | 1.00 |  |  | 1.00 |  |  | 1.00 |  |  | 1.00 |  |  | 2270, 3233 |
| Yes | 6.95 | (2.85-16.92) |  | 1.68 | (0.90-3.15) |  | 1.29 | (0.42-3.98) |  | 3.20 | (1.65-6.19) |  | 676, 988 |
| **Partner shares same sexual likes and dislikes** |  |  | 0.0003 |  |  | 0.0204 |  |  | 0.0033 |  |  | 0.0053 |  |
| No | 1.00 |  |  | 1.00 |  |  | 1.00 |  |  | 1.00 |  |  | 2650, 3803 |
| Yes | 5.21 | (2.12-12.82) |  | 2.39 | (1.15-4.99) |  | 5.15 | (1.73-15.36) |  | 2.81 | (1.36-5.81) |  | 296, 418 |
| **INDIVIDUAL VULNERABILITY** |  |  |  |  |  |  |  |  |  |  |  |  |  |
| **Felt anxious during sex** |  |  | <0.0001 |  |  | <0.0001 |  |  | <0.0001 |  |  | <0.0001 |  |
| No | 1.00 |  |  | 1.00 |  |  | 1.00 |  |  | 1.00 |  |  | 4548, 5651 |
| Yes | 9.18 | (4.27-19.74) |  | 8.47 | (4.91-14.59) |  | 9.94 | (4.05-24.39) |  | 5.74 | (3.26-10.10) |  | 292, 324 |
| **Uncomfortably dry vagina** |  |  | - |  |  | - |  |  | - |  |  | - |  |
| No | - | - |  | - | - |  | - | - |  | - | - |  | - |
| Yes | - | - |  | - | - |  | - | - |  | - | - |  | - |
| **Experienced non-volitional sex, ever** |  |  | - |  |  | - |  |  | - |  |  | - |  |
| No | - | - |  | - | - |  | - | - |  | - | - |  | - |
| Yes | - | - |  | - | - |  | - | - |  | - | - |  | - |
| **Unemployed last week** |  |  | 0.6104 |  |  | 0.1164 |  |  | 0.2006 |  |  | 0.5689 |  |
| No | 1.00 |  |  | 1.00 |  |  | 1.00 |  |  | 1.00 |  |  | 3277, 4307 |
| Yes | 1.24 | (0.54-2.84) |  | 1.56 | (0.90-2.71) |  | 1.93 | (0.71-5.29) |  | 1.19 | (0.66-2.15) |  | 1560, 1666 |
| **Current depression (PHQ-2)** |  |  | <0.0001 |  |  | 0.0007 |  |  | <0.0001 |  |  | 0.0001 |  |
| No | 1.00 |  |  | 1.00 |  |  | 1.00 |  |  | 1.00 |  |  | 4384, 5472 |
| Yes | 12.01 | (5.54-26.04) |  | 3.05 | (1.61-5.80) |  | 8.50 | (3.60-20.05) |  | 3.43 | (1.88-6.26) |  | 449, 495 |
| **CULTURAL/RELIGIOUS FACTORS** |  |  |  |  |  |  |  |  |  |  |  |  |  |
| **Religion important and practiced regularly** |  |  | 0.5460 |  |  | 0.9003 |  |  | 0.2854 |  |  | 0.4590 |  |
| No | 1.00 |  |  | 1.00 |  |  | 1.00 |  |  | 1.00 |  |  | 4481, 5453 |
| Yes | 0.64 | (0.15-2.77) |  | 0.95 | (0.41-2.18) |  | 1.97 | (0.57-6.85) |  | 1.42 | (0.56-3.60) |  | 349, 506 |
| **Sex without love OK** |  |  | 0.4846 |  |  | 0.2887 |  |  | 0.0152 |  |  | 0.8326 |  |
| No | 1.00 |  |  | 1.00 |  |  | 1.00 |  |  | 1.00 |  |  | 1586, 2097 |
| Yes | 1.34 | (0.59-3.04) |  | 1.33 | (0.78-2.26) |  | 0.33 | (0.13-0.80) |  | 0.94 | (0.54-1.64) |  | 3233, 3852 |
| **People are under pressure to have sex** |  |  | 0.9621 |  |  | 0.3016 |  |  | 0.0231 |  |  | 0.9643 |  |
| No | 1.00 |  |  | 1.00 |  |  | 1.00 |  |  | 1.00 |  |  | 1737, 2176 |
| Yes | 0.98 | (0.42-2.27) |  | 1.31 | (0.78-2.21) |  | 4.66 | (1.24-17.55) |  | 0.99 | (0.57-1.72) |  | 3039, 3708 |
| **MEDICAL FACTORS** |  |  |  |  |  |  |  |  |  |  |  |  |  |
| **Number of self-reported chronic conditions** |  |  | 0.0060 |  |  | 0.0049 |  |  | 0.0210 |  |  | 0.1915 |  |
| 0 | 1.00 |  |  | 1.00 |  |  | 1.00 |  |  | 1.00 |  |  | 3456, 3996 |
| 1 | 3.54 | (1.18-10.67) |  | 1.28 | (0.64-2.54) |  | 3.55 | (1.08-11.72) |  | 0.98 | (0.50-1.95) |  | 920, 1302 |
| 2+ | 7.38 | (2.18-24.99) |  | 3.04 | (1.48-6.25) |  | 5.88 | (1.65-20.97) |  | 2.27 | (0.89-5.82) |  | 464, 678 |
| **Health affecting sexual activity or enjoyment** |  |  | <0.0001 |  |  | <0.0001 |  |  | 0.0017 |  |  | 0.0002 |  |
| No | 1.00 |  |  | 1.00 |  |  | 1.00 |  |  | 1.00 |  |  | 4170, 5061 |
| Yes | 11.85 | (4.53-30.98) |  | 14.20 | (8.05-25.05) |  | 4.94 | (1.82-13.36) |  | 3.00 | (1.68-5.35) |  | 656, 898 |
| **Medication that affected sexual activity last year** |  |  | <0.0001 |  |  | <0.0001 |  |  | 0.0195 |  |  | 0.2708 |  |
| No | 1.00 |  |  | 1.00 |  |  | 1.00 |  |  | 1.00 |  |  | 4492, 5513 |
| Yes | 11.97 | (4.92-29.09) |  | 5.86 | (3.40-10.07) |  | 4.49 | (1.28-15.81) |  | 1.62 | (0.69-3.83) |  | 332, 444 |

|  | **Women** | | | | | | | | | |
| --- | --- | --- | --- | --- | --- | --- | --- | --- | --- | --- |
|  | **Lacked interest/arousal in having sex** | | | **Reached orgasm too slowly/not at all** | | | **Physical pain as a result of sex** | | |  |
|  | **aAOR** | **95% C.I.** | **p-value** | **aAOR** | **95% C.I.** | **p-value** | **aAOR** | **95% C.I.** | **p-value** | **Denominators** |
| **Age group** |  |  | 0.6687 |  |  | 0.0157 |  |  | 0.0182 |  |
| 16-24 | 1.00 |  |  | 1.00 |  |  | 1.00 |  |  | 1662, 923 |
| 25-34 | 1.32 | (0.56-3.10) |  | 0.79 | (0.51-1.24) |  | 1.19 | (0.69-2.07) |  | 2236, 1246 |
| 35-44 | 0.54 | (0.14-2.11) |  | 0.42 | (0.22-0.80) |  | 0.91 | (0.46-1.76) |  | 1050, 1290 |
| 45-54 | 1.14 | (0.39-3.31) |  | 0.73 | (0.40-1.32) |  | 0.69 | (0.33-1.45) |  | 871, 1186 |
| 55-64 | 1.49 | (0.51-4.37) |  | 0.57 | (0.28-1.18) |  | 2.39 | (1.26-4.56) |  | 569, 755 |
| 65-74 | 1.00 | - |  | 0.07 | (0.01-0.49) |  | 0.67 | (0.22-2.05) |  | 281, 355 |
| **PARTNER AND RELATIONSHIP FACTORS** |  |  |  |  |  |  |  |  |  |  |
| **Partner had sexual difficulties past year** |  |  | 0.5391 |  |  | 0.0007 |  |  | 0.0910 |  |
| No | 1.00 |  |  | 1.00 |  |  | 1.00 |  |  | 3726, 3498 |
| Yes | 1.40 | (0.48-4.06) |  | 2.77 | (1.54-4.98) |  | 1.69 | (0.92-3.10) |  | 649, 719 |
| **Always easy to talk about sex with partner** |  |  | 0.7565 |  |  | 0.1297 |  |  | 0.0011 |  |
| Yes | 1.00 |  |  | 1.00 |  |  | 1.00 |  |  | 1746, 1451 |
| Else | 0.87 | (0.36-2.08) |  | 1.50 | (0.89-2.54) |  | 2.30 | (1.40-3.79) |  | 4907, 4289 |
| **Partner shares same interest level in sex** |  |  | <0.0001 |  |  | <0.0001 |  |  | <0.0001 |  |
| No | 1.00 |  |  | 1.00 |  |  | 1.00 |  |  | 3211, 3064 |
| Yes | 13.79 | (5.15-36.94) |  | 5.47 | (3.21-9.30) |  | 3.78 | (2.32-6.16) |  | 1166, 1155 |
| **Partner shares same sexual likes and dislikes** |  |  | <0.0001 |  |  | <0.0001 |  |  | 0.0012 |  |
| No | 1.00 |  |  | 1.00 |  |  | 1.00 |  |  | 4079, 3908 |
| Yes | 7.68 | (3.29-17.95) |  | 5.33 | (3.03-9.40) |  | 2.86 | (1.51-5.39) |  | 297, 310 |
| **INDIVIDUAL VULNERABILITY** |  |  |  |  |  |  |  |  |  |  |
| **Felt anxious during sex** |  |  | <0.0001 |  |  | <0.0001 |  |  | <0.0001 |  |
| No | 1.00 |  |  | 1.00 |  |  | 1.00 |  |  | 6264, 5453 |
| Yes | 27.06 | (12.80-57.22) |  | 10.85 | (6.87-17.12) |  | 10.33 | (6.25-17.06) |  | 405, 302 |
| **Uncomfortably dry vagina** |  |  | <0.0001 |  |  | <0.0001 |  |  | <0.0001 |  |
| No | 1.00 |  |  | 1.00 |  |  | 1.00 |  |  | 5920, 5010 |
| Yes | 7.21 | (3.24-16.03) |  | 5.29 | (3.47-8.05) |  | 11.23 | (7.36-17.13) |  | 749, 746 |
| **Experienced non-volitional sex, ever** |  |  | 0.0006 |  |  | <0.0001 |  |  | 0.0003 |  |
| No | 1.00 |  |  | 1.00 |  |  | 1.00 |  |  | 5815, 5055 |
| Yes | 3.78 | (1.78-8.04) |  | 3.55 | (2.27-5.56) |  | 2.48 | (1.52-4.05) |  | 684, 579 |
| **Unemployed last week** |  |  | 0.9452 |  |  | 0.4312 |  |  | 0.4501 |  |
| No | 1.00 |  |  | 1.00 |  |  | 1.00 |  |  | 4001, 3597 |
| Yes | 1.03 | (0.50-2.12) |  | 0.85 | (0.57-1.28) |  | 1.18 | (0.77-1.79) |  | 2662, 2152 |
| **Current depression (PHQ-2)** |  |  | 0.0003 |  |  | <0.0001 |  |  | <0.0001 |  |
| No | 1.00 |  |  | 1.00 |  |  | 1.00 |  |  | 5885, 5149 |
| Yes | 3.71 | (1.82-7.56) |  | 3.31 | (2.18-5.03) |  | 2.85 | (1.79-4.54) |  | 780, 602 |
| **CULTURAL/RELIGIOUS FACTORS** |  |  |  |  |  |  |  |  |  |  |
| **Religion important and practiced regularly** |  |  | 0.9769 |  |  | 0.3471 |  |  | 0.1962 |  |
| No | 1.00 |  |  | 1.00 |  |  | 1.00 |  |  | 6047, 5100 |
| Yes | 0.98 | (0.23-4.19) |  | 0.65 | (0.27-1.59) |  | 0.57 | (0.24-1.34) |  | 609, 644 |
| **Sex without love OK** |  |  | 0.6992 |  |  | 0.0137 |  |  | 0.8295 |  |
| No | 1.00 |  |  | 1.00 |  |  | 1.00 |  |  | 3291, 2968 |
| Yes | 1.16 | (0.55-2.45) |  | 1.68 | (1.11-2.52) |  | 0.95 | (0.62-1.47) |  | 3347, 2761 |
| **People are under pressure to have sex** |  |  | 0.2501 |  |  | 0.0854 |  |  | 0.1539 |  |
| No | 1.00 |  |  | 1.00 |  |  | 1.00 |  |  | 1761, 1485 |
| Yes | 1.91 | (0.64-5.72) |  | 1.57 | (0.94-2.63) |  | 1.47 | (0.87-2.51) |  | 4817, 4185 |
| **MEDICAL FACTORS** |  |  |  |  |  |  |  |  |  |  |
| **Number of self-reported chronic conditions** |  |  | 0.0529 |  |  | 0.0003 |  |  | <0.0001 |  |
| 0 | 1.00 |  |  | 1.00 |  |  | 1.00 |  |  | 4357, 3536 |
| 1 | 2.90 | (1.22-6.87) |  | 2.05 | (1.30-3.25) |  | 1.79 | (1.09-2.93) |  | 1544, 1405 |
| 2+ | 2.72 | (0.78-9.53) |  | 2.98 | (1.66-5.34) |  | 4.15 | (2.38-7.25) |  | 767, 814 |
| **Health affecting sexual activity or enjoyment** |  |  | <0.0001 |  |  | <0.0001 |  |  | <0.0001 |  |
| No | 1.00 |  |  | 1.00 |  |  | 1.00 |  |  | 5515, 4712 |
| Yes | 8.90 | (4.32-18.30) |  | 4.97 | (3.31-7.45) |  | 10.85 | (7.01-16.82) |  | 1147, 1038 |
| **Medication that affected sexual activity last year** |  |  | <0.0001 |  |  | <0.0001 |  |  | 0.0001 |  |
| No | 1.00 |  |  | 1.00 |  |  | 1.00 |  |  | 6170, 5318 |
| Yes | 7.05 | (3.26-15.24) |  | 6.63 | (4.30-10.22) |  | 2.93 | (1.73-4.96) |  | 492, 431 |
